# Relevant microclimate for determining the development rate of malaria mosquitoes and possible implications of climate change

**DOI:** 10.1186/1475-2875-9-196

**Published:** 2010-07-09

**Authors:** Krijn P Paaijmans, Susan S Imbahale, Matthew B Thomas, Willem Takken

**Affiliations:** 1 Laboratory of Entomology, Wageningen University, PO Box 8031, 6700EH Wageningen, The Netherlands; 2 Meteorology and Air Quality, Wageningen University, PO Box 47, 6700AA Wageningen, The Netherlands; 3 Center for Infectious Disease Dynamics and Department of Entomology, Pennsylvania State University, University Park, PA16802, USA

## Abstract

**Background:**

The relationship between mosquito development and temperature is one of the keys to understanding the current and future dynamics and distribution of vector-borne diseases such as malaria. Many process-based models use mean air temperature to estimate larval development times, and hence adult vector densities and/or malaria risk.

**Methods:**

Water temperatures in three different-sized water pools, as well as the adjacent air temperature in lowland and highland sites in western Kenya were monitored. Both air and water temperatures were fed into a widely-applied temperature-dependent development model for *Anopheles gambiae *immatures, and subsequently their impact on predicted vector abundance was assessed.

**Results:**

Mean water temperature in typical mosquito breeding sites was 4-6°C higher than the mean temperature of the adjacent air, resulting in larval development rates, and hence population growth rates, that are much higher than predicted based on air temperature. On the other hand, due to the non-linearities in the relationship between temperature and larval development rate, together with a marginal buffering in the increase in water temperature compared with air temperature, the relative increases in larval development rates predicted due to climate change are substantially less.

**Conclusions:**

Existing models will tend to underestimate mosquito population growth under current conditions, and may overestimate relative increases in population growth under future climate change. These results highlight the need for better integration of biological and environmental information at the scale relevant to mosquito biology.

## Background

The last few decades have seen a marked resurgence of malaria in the East African highlands [reviewed in [1]]. The reasons for this increase in seasonal malaria are not yet fully resolved but one factor that has been widely debated is the possible influence of regional warming due to climate change [2-7]. Amongst the studies supporting a climate-driven response, Pascual *et al *[3] used a temperature-dependent population dynamic model to demonstrate that a small change in mean ambient air temperature of just 0.5°C could translate into a 30-100% increase in mosquito abundance [see also [8]]. One of the reasons for this pronounced biological amplification is the strongly non-linear effects of temperature on mosquito larval development rate around the relatively cool ambient temperatures typical for highland areas.

More generally, the relationship between mosquito biology and temperature is central to numerous studies exploring the temporal and/or spatial patterns of malaria risk [3,9-13]. A feature of nearly all such studies is the use of mean ambient air temperature to drive the relevant rate processes. While this might be appropriate for processes relating to transmission by the adult mosquito [but see also [14]], the immature stages of malaria mosquitoes, such as *Anopheles gambiae*, inhabit aquatic environments such as small, transient, sunlit pools [e.g. [15]].

The aim of the current study was to examine how ambient temperature relates to the water temperature in larval habitats in lowland and highland areas of western Kenya and to explore the biological effects of these temperature relations for larval development. The study re-analyses data from a previous investigation by Paaijmans *et al *[16] conducted at a lowland site (this earlier study, conducted in Kisian, focussed on modelling water temperatures using energy budgets) and adds new empirical data for two highland sites, Lyanaginga and Fort Ternan. The study also extends analysis to explore conditions at Kericho, a cooler highland area in western Kenya that has been at the centre of the debate on whether climate change has impacted on recent malaria dynamics [1-3,17-21]. The aim is not to question the relationship between temperature and *Anopheles *larval development *per se *but rather to ask: (i) whether air temperature provides an appropriate direct measure for determining development rate of larvae in aquatic habitats; and (ii) whether the absolute and relative increases in larval development rate predicted from increases in mean air temperature are the same when the relationship between air and water temperature is considered. The answer in both cases is no, raising concerns over the broad application of air temperature for exploring mosquito dynamics and malaria risk, both now and under future climate change scenarios.

## Methods

### Microclimate data

Air temperature (ventilated HMP45C probe, Campbell Scientific, UK; 2 m height) and rainfall (ARG100 Tipping Bucket Raingauge, Campbell Scientific, UK) were measured at two highland sites in western Kenya: Lyanaginga (Vihiga District; 0°1'0.27"N 34°39'34.42"E, 1,542 m) and Fort Ternan (Kericho district; 0°12'7.74"S; 35°20'37.57"E, altitude 1,552 m).

Adjacent to the weather stations, three different-sized artificial water pools were created to simulate natural mosquito larval habitats. Pools were made by digging holes in the ground: a small-sized water pool (diameter 0.16 m × water depth 0.04 m, volume 0.8 L), a medium-sized pool (0.32 m × 0.16 m, 12.9 L) and a large-sized pool (0.96 m × 0.32 m, 231.6 L). These pool dimensions were selected to represent an animal hoof print, a small rain puddle and a burrow pit, respectively, all typical *An. gambiae *breeding habitats [15,22]. Pools were lined with clear semi-transparent plastic (0.13 mm thick), which was pressed tightly against the soil to maximize plastic-soil contact, and filled with clear tap water to within 10 mm of the brim. A uniform water level was maintained by adding or removing water each morning. Experimental areas were vegetated by grass, which was kept short. Water temperature was measured in the centre of each water pool using glass bead thermistors (NTC BEAD 4K7, Thermometrics, USA) positioned near the air-water interface to provide water temperature measurements to a depth of 1-5 mm. The reader is referred to Paaijmans *et al *[16] for more detailed information and images of the thermistors and floating equipment. Meteorological data were totaled (rainfall) or averaged (temperature) over 15 min intervals using a CR1000 Datalogger (Campbell Scientific, U.K.). Data were recorded between 27 April and 27 May 2006, a period during the 'long rains' in which populations of *An. gambiae *build up rapidly. Measures of air temperature were compared with the adjacent water temperatures using simple linear regression (SPPS v. 17, SPSS Inc., Chicago, IL).

In addition to these highland data, a reanalysis of equivalent data recorded from similar artificial ponds in a lowland area of western Kenya [16] is also presented. These data were collected in Kisian (0°4'36.23"S; 34°40'34.78"E, altitude 1,126 m) between 16 March and 5 May 2005. The experimental methods used were broadly consistent with those described above [full details are presented in [16]]. Note for both lowland and highland data sets, the number of days of air temperature recordings slightly exceeds the number of days of water temperature recordings, due to occasional maintenance of the water pool set-up.

### Mosquito population growth rates

To determine the effects of mean temperature (*T*) on larval development period (*d*) of *An. gambiae *the widely-applied model presented in Craig *et al *[9] was used, where *d(T) *= 1/(0.00554*T *- 0.06737). Development period was estimated for both air temperature and the corresponding water temperature for each water pool in each location.

The effects of any changes in larval development period on population growth rate were explored by calculating the intrinsic rate of increase, *r*, using the analytical approximation *r *= ln *R*_0_/*G*, where *R*_*0 *_the net reproductive rate and *G *is mean length of a generation [23]. Parameter estimates for *G *and *R*_*0 *_were derived from the study of Afrane *et al *[24] who measured adult longevity and daily reproductive fitness for *An. gambiae *at a lowland and highland site in Western Kenya during the rainy season when mean air temperatures were almost identical to those of the current study. Net reproductive rate (*R*_*0*_) was estimated directly by Afrane *et al *[24] with values of *R*_*0 *_= 346.0 and *R*_*0 *_= 434.8 for the lowland and highland sites, respectively. Mean length of a generation was calculated as the median time for adult reproduction (24.7 and 20.7 days for the lowland and highland sites, respectively, data derived from Afrane *et al *[24]) plus the relevant duration of larval development from the current study to obtain the mean length of a complete generation (i.e. including the subimagal stages).

### Potential impact of climate change

To examine the effect of global warming on larval development, two climate change scenarios were considered. First, using the data from the highland sites the retrospective effects of a 1°C rise in temperature suggested to have occurred in the Kenyan highlands since the 1970's [25] were investigated. Then a longer-term prospective scenario with increases in mean monthly temperature of 3.2°C was considered, corresponding to the median increase in terrestrial temperature predicted by the IPCC for the months March-May in East-Africa by 2100 [26].

Subsequently, changes in mosquito population growth rates and mosquito abundance over time were examined for each of these climate scenarios. While warming over these temperature ranges is expected to affect adult mosquito traits that determine the baseline net reproductive rate (e.g. size, feeding, fecundity, period of gonotrophic cycle, flight potential, host and oviposition-site searching efficiency etc.), it is not known how temperature affects all of these traits, nor the net effect of simultaneous impacts across multiple traits and so for simplicity the analyses consider effects via changes in immature development only. Accordingly, any predicted changes in intrinsic rate of increase are likely to be conservative.

## Results and discussion

### Microclimate

**Figure 1:**
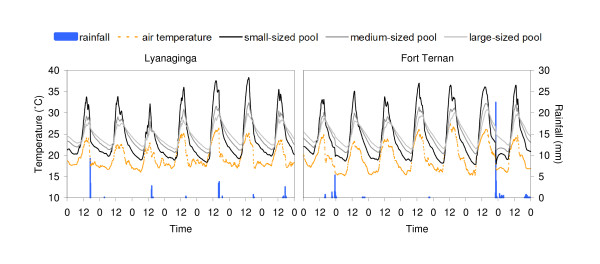
**Water temperature dynamics near the air-water interface of three different-sized pools, air temperature and rainfall during a representative 7 day period in Lyanaginga and Fort Ternan, two highland areas in western Kenya**.

Water temperatures in Lyanaginga and Fort Ternan were generally higher than corresponding air temperatures throughout most of the day, with mean water temperature exceeding mean air temperature by 4.5 to 5.8°C over the study period, depending on pool size and location (Figure 1, Table 1). Similar but slightly less pronounced patterns (3.7-4.3°C) were also found in Kisian [Table 1, [16]].

**Table 1:** Mean air and water temperatures in one lowland and two highland sites in western Kenya

	Air temperature (°C)	Water temperature (°C)		
		small-sized pool	medium-sized pool	large-sized pool
Kisian (LL)	23.4 ± 0.2^51^	27.1 ± 0.2 ^38^	27.6 ± 0.1 ^39^	27.7 ± 0.1 ^40^
Lyanaginga (HL)	19.4 ± 0.1^31^	23.9 ± 0.2 ^30^	24.1 ± 0.2 ^30^	24.6 ± 0.1 ^30^
Fort Ternan (HL)	19.3 ± 0.1^31^	24.3 ± 0.2 ^15^*	24.7 ± 0.1 ^29^	25.1 ± 0.1 ^30^

Standard error of the mean is given, LL indicates lowland area, HL highland area. Numbers in superscript indicate the number of days used in the analysis; the asterisk indicates a low number due to temporary failure of the thermistors during short periods in the early mornings, when the minimum water temperature is normally recorded

To what extend the plastic boundary layer might have influenced water temperature in the artificial pools remains unclear. A previous study showed that the thickness of the plastic foil used in the artificial pools did not interfere with the soil heat flux, as the heat resistance of plastic is low, but that a small amount of the incoming solar radiation is reflected by the plastic and therefore, not absorbed by the soil [see [16]]. However, several field studies have reported very similar mean air and water temperatures to those presented here, in *An. gambiae *larval habitats in both highland and lowland areas in western Kenya [27-29]. As such, the authors are confident that the water temperatures in the experimental pools are representative of natural *An. gambiae *breeding habitats of equivalent dimensions.

**Figure 2:**
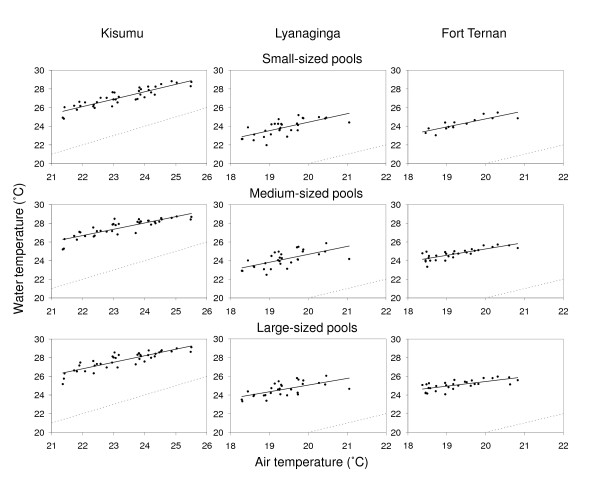
**Relationship between the daily mean air and daily mean water temperature, as observed in a lowland site (Kisian) and in two highland sites (Lyanaginga and Fort Ternan) in western Kenya**. Black lines represent regression lines, grey dotted lines the 1:1 (air:water temperature) ratio.

The differences between air and water temperatures are captured in the regression analyses, which demonstrate differences in both slope and intercept between water and air temperatures (Figure 2; Table 2). Due to these differences, it is clear that changes in air temperature do not translate directly to changes in water temperature either in absolute or relative terms. A change of 1°C in mean air temperature, for example, results in changes in mean water temperature from 0.9 to as low as 0.5°C, depending on size and location of the pool.

**Table 2:** Linear regressions of the relation between mean air (independent variable) and mean water temperature (dependent variable) in one lowland (LL) and two highland sites (HL) in western Kenya.

	*a*	*b*	***R***^***2***^	*ANOVA*
Small-sized pool				
*Kisian (LL)*	.794	8.646	.803	F_1,37 _= 147, *P *< 0.001
*Lyanaginga (HL)*	.911	6.209	.487	F_1,29 _= 26.6, *P *< 0.001
*Fort Ternan (HL)*	.856	7.654	.719	F_1,14 _= 33.3, *P *< 0.001
				
Medium-sized pool				
*Kisian (LL)*	.677	11.802	.745	F_1,38 _= 108, *P *< 0.001
*Lyanaginga (HL)*	.846	7.745	.407	F_1,29 _= 19.2, *P *< 0.001
*Fort Ternan (HL)*	.677	11.715	.639	F_1,28 _= 47.8, *P *< 0.001
				
Large-sized pool				
*Kisian (LL)*	.700	11.400	.743	F_1,39 _= 110, *P *< 0.001
*Lyanaginga (HL)*	.710	10.852	.405	F_1,29 _= 19.0, *P *< 0.001
*Fort Ternan (HL)*	.503	15.395	.483	F_1,29 _= 26.1, *P *< 0.001

*a *slope; *b *intercept; LL lowland area; HL highland area

Empirical data and regression lines are plotted in Figure 2.

#### Mosquito population growth rates

**Figure 3:**
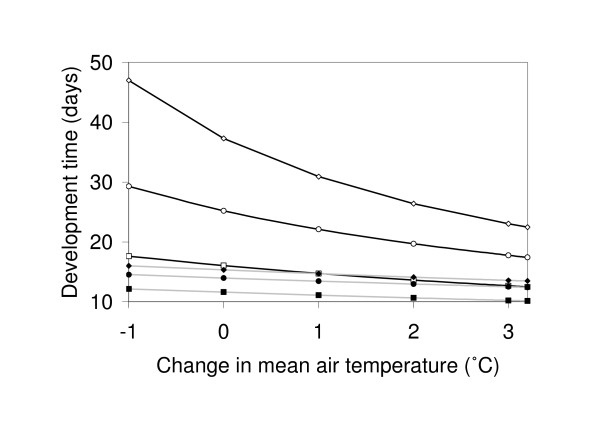
**Larval development times of *Anopheles gambiae*, as predicted with air (open symbols) and water temperatures (closed symbols) for Kisian (squares), a lowland area in western Kenya and Fort Ternan (circles) and Kericho (diamonds), two highland areas in western Kenya, considering historical (negative values on x-axis), present-day (0°C), or future (positive values on x-axis) climate conditions**. For illustrative purposes, results are presented for the large-sized pools only (patterns are qualitatively similar for other pool sizes).

The observed temperature differences lead to large differences in estimated development times, with higher mean water temperatures shortening larval duration by 4.0-4.4 days (equivalent to 25-28%) in Kisian, and by 9.7-11.2 days (equivalent to 39-45%) in Lyanaginga and Fort Ternan, compared with estimates based on the mean air temperatures (Figure 3). The immature development times that are derived using aquatic temperatures in the current study are consistent with the observed larval development times in several field studies in western Kenya [28-31].

**Figure 4:**
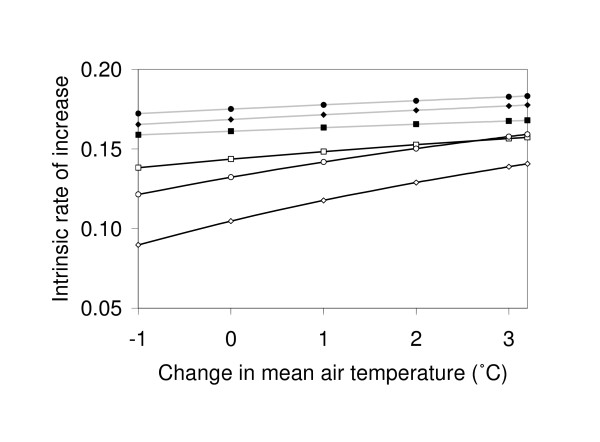
**Intrinsic rates of increase of *Anopheles gambiae *populations, as predicted with air (open symbols) and water temperatures (closed symbols) for Kisian (squares), a lowland area in western Kenya and Fort Ternan (circles) and Kericho (diamonds), two highland areas in western Kenya, considering historical (negative values on x-axis), present-day (0°C), or future (positive values on x-axis) climate conditions.** Again, results are presented for the large-sized pools only. Note that only an effect of climate change on larval development time is assumed (see methods).

These more rapid development rates translate into intrinsic rates of increase 11-12% higher in the lowland area and 27-32% higher in the highland areas, compared with estimates based on mean air temperatures (Figure 4). Quantifying what these differences in rates of increase mean in terms of actual abundance is complicated since many factors determine overall population dynamics. However, as an illustrative case for the highlands, if one assumes simple exponential growth such that the number (*N*) of mosquitoes is approximated by *N *= *N*_0_*e*^*rt *^[23], and further assumes a season length of 5 months (malaria in the East African Highlands is not holoendemic [19] and entomological surveys indicate the principal mosquito vectors are largely restricted to a 4-5-month window [32,33]) one can find, for example, that air temperature predicts potential populations some 540-fold lower than those expected based on water temperature in the large pools in Fort Ternan (note in this instance it is legitimate to consider effects via immature development alone since the empirical estimates of present-day net reproductive rate apply equally to all adult mosquitoes).

### Potential impact of climate change

Subtracting 1°C from current air temperatures at the highland sites suggests that recent warming in the East-African highlands has shortened larval developmental periods by approximately 4.1 days in Lyanaginga and Fort Ternan (Figure 3), leading to an increase in present-day intrinsic rates of population growth of 9% (Figure 4). However, this same climate signal yields very different results when considering water temperature. First, because water temperatures are higher than equivalent air temperatures, the baseline larval development and population growth rates are themselves much higher. Second, at these higher temperatures, due to the saturating nature of the temperature-development relationship [see [9]], the effect of a small rise in temperature on development period is much less pronounced. Finally, the actual increase in water temperature is expected to be less than 1°C since the slopes of the regression lines describing the relationship between water and air temperature are <1 (Figure 2, Table 2). Combined, these effects mean that the increases in population growth rate due to effects of recent warming on larval development are expected to be between 2 and 4% depending on size and location of the pool (Figure 4).

A similar pattern emerges from analysis of future climate change. Adding the projected increase of 3.2°C in temperature to current air temperatures dramatically shortens larval development period and increases population growth rate (Figures 3 and 4). Again, however, the magnitude of these effects is greatly reduced when the relationship between air and water temperature is taken into account; instead of decreases in larval development times of 7.7-7.8 days in the highland and 3.6 days in the lowland areas, development times are predicted to shorten by only 1.5-3.1 days and 1.4-1.8 days, respectively, depending on pool size and location (Figure 3). As a consequence, the increases in population growth rates are predicted to be in the order of 5-9% in the highland areas and 4-5% in the lowland area rather than 17% and 9%, respectively (Figure 4). Essentially, although warming is expected to increase growth rate via effects on larval development, the relative change (indicated by the slopes of the lines in Figure 4) is expected to be much less when the influence of water temperature is taken into account.

### Kericho

Based on the temperature data presented by Pascual *et al *[25], the average air temperature during the 4-5 month annual transmission season in recent years is estimated to be 17°C (the average air temperature tends to be higher during the malaria transmission season, compared to the annual mean). As there are no empirical data on water temperatures in mosquito breeding sites in Kericho to our knowledge, the simple linear regressions between the air temperature and the water temperatures obtained for Fort Ternan (~25 km away) (Table 2) were used to estimate water temperatures in Kericho, using this air temperature of 17°C.

With these cool mean air temperatures, the differences between the observed air and extrapolated water temperatures result in large differences in estimated development times, with the higher mean water temperatures shortening larval duration by 19-22 days (equivalent to 52-59%) depending on pool size, compared with estimates based on the mean air temperatures (see Figure 3 for illustrative results for the large pools). These more rapid development rates translate into intrinsic rates of increase 50-61% higher, compared with estimates based on mean air temperatures (Figure 4).

With respect to climate change, the results for Kericho broadly follow those of the other highland sites described above, although with slightly more marked effects. For the recent warming of 1°C, for example, the increase in air temperature is predicted to reduce larval duration from 47 to 37 days, whereas with water temperature (large pool size), larval duration is reduced from 16 to 15 days (Figure 3). These developmental times result in increases in population growth rates from 0.090 to 0.105 (an increase of 17%) compared with 0.165 to 0.169 (an increase of 2%), respectively. Following similar assumptions to those above (including caveats regarding exponential growth and what factors ultimately regulate populations in nature), such differences in intrinsic rates of increase lead to 9.1-fold increase in potential mosquito abundance by the end of the malaria transmission season based on historic changes in air temperature, but only 1.6-fold when corrected for more modest changes in water temperature (albeit with much higher absolute numbers because the baseline rate of increase is itself higher).

Another potentially important malaria vector species in Kericho, *Anopheles funestus *[32,33], generally breeds in larger permanent or semi-permanent swamps or pools [34]. We are not aware of studies recording continued water temperature readings of such mosquito breeding sites. However, a study assessing water temperatures in The Wellcome Dam in Kenya (a large permanent pool with dimensions 85 × 152 × 152 m, at an altitude of 1,661 m), reported qualitatively similar results to those reported here, with average weekly air temperatures frequently being >4°C lower than the average weekly water temperatures [35]. It appears, therefore, that discrepancies between air and water temperatures will likely apply to breeding habitats of mosquitoes other than *An. gambiae*.

## Conclusions

Given the fundamental fact that mosquito larvae live in aquatic and not terrestrial habitats, our results suggest that although widely used [3,9-12], air temperature alone does not provide an appropriate variable for estimating immature mosquito development or for setting threshold temperatures. The analyses in the present paper suggest that use of air temperature rather than water temperature will tend to under-estimate current population growth rates, while strongly over-estimating the impact of warming on population growth rates. This latter effect applies to the long-term increases in temperature projected for the current century, as well as the purported historical warming trend in the East African highlands to date. While many factors beyond just larval developmental period combine to determine overall mosquito population size and intensity of malaria transmission [e.g. [36]], the potential for direct biological amplification of mosquito abundance via more rapid larval development due to climate change appears overstated. Important to note is that these findings do not argue against an impact of climate change on the dynamics and distribution of mosquitoes and malaria, but rather that the relative contribution from increases in mosquito population growth rates will tend to be smaller than expected. The current study highlights a need to consider environmental factors in a way relevant to the mosquito and cautions against the direct application of air temperature when modeling larval population dynamics. The strong and significant relationships between water temperature and air temperature suggest the potential to predict mean water temperature quite accurately using the mean air temperature. However, more studies are required to examine the exact relationships in a diversity of natural pool types in different environmental settings. Application of the representative regressions could enable better estimates of *An. gambiae *larval development with respect to air temperature data, leading to improved understanding of the dynamics of mosquitoes and ultimately malaria under future climate change.

## Conflict of interest statement

The authors declare that they have no competing interests.

## Authors' contributions

KPP designed the experiment. KPP and SSI collected the data. All authors participated in the interpretation of data, in the preparation of the manuscript and approved the final version.
